# Arrhythmic risk stratification in post-myocardial infarction patients with preserved ejection fraction: long-term outcomes from the PRESERVE EF study

**DOI:** 10.3389/fcvm.2026.1787632

**Published:** 2026-07-03

**Authors:** Ioannis Doundoulakis, Dimitris Tsiachris, Petros Arsenos, Athanasios Kordalis, Christos-Konstantinos Antoniou, Konstantinos Vlachos, Stergios Soulaidopoulos, Aggeliki Laina, Emmanuel Kanoupakis, Polychronis Dilaveris, Theofilos M. Kolettis, Konstantinos Trachanas, Iosif Xenogiannis, Panagiotis Korantzopoulos, Skevos Sideris, Nikolaos Fragakis, Vassilios P. Vassilikos, Konstantinos Tsioufis, Konstantinos A. Gatzoulis

**Affiliations:** 1 First University Department of Cardiology, National and Kapodistrian University of Athens, Athens, Greece; 2 Heart Rhythm Management Centre, Universitair Ziekenhuis Brussel, Heart Rhythm Research Brussels, Postgraduate Program in Cardiac Electrophysiology and Pacing, Vrije Universiteit Brussel, European Reference Networks Guard-Heart, Laarbeeklaan, Brussels, Belgium; 3 IHU Liryc, Electrophysiology and Heart Modeling Institute, Bordeaux, France; 4 Department of Cardiology, Haut-Leveque University Hospital, Bordeaux, France; 5 Department of Cardiology, University Hospital of Heraklion, University of Crete, Heraklion, Greece; 6 First Cardiology Division, University Hospital of Ioannina, University of Ioannina, Ioannina, Greece; 7 State Department of Cardiology, “Hippokration” General Hospital of Athens, Athens, Greece; 8 Second Cardiology Department, National and Kapodistrian University of Athens, Attikon Hospital, Athens, Greece; 9 Second Department of Cardiology, Hippokration General Hospital, Aristotle University of Thessaloniki, Thessaloniki, Greece; 10 Third Cardiology Department, School of Medicine, Aristotle University of Thessaloniki, Hippokrateion General Hospital, Thessaloniki, Greece

**Keywords:** electrophysiology study, myocardial infarction, preserved ejection fraction, programmed ventricular stimulation, risk stratification

## Abstract

**Introduction:**

The PRESERVE EF study proposed a two-step algorithm for risk stratification in post-myocardial infarction (MI) patients with mid-range and preserved left ventricular ejection fraction (LVEF). This method assessed the performance of a two-step, programmed ventricular stimulation (PVS)-inclusive approach in identifying high-risk post-MI patients with LVEF ≥40%. This report presents findings from the 8-year follow-up.

**Methods:**

The primary endpoint was the occurrence of a major arrhythmic event, defined as sustained ventricular tachycardia/fibrillation, appropriate implantable cardioverter-defibrillator (ICD) activation, or sudden cardiac death (SCD). We included a total of 575 consecutive patients (mean age 57 years, LVEF 50.8%). Of them, 204 (35.5%) had at least one positive non-invasive risk factor. Forty-one of 152 patients undergoing PVS were inducible; 37 (90.2%) of them received an ICD.

**Results:**

During a mean follow-up of 106 ± 14.5 months, no SCDs were observed, while 12 ICDs (the major arrhythmic event prevalence in patients with ICD implantation reaching 29.3%) were appropriately activated. The updated performance metrics of the proposed approach were as follows: sensitivity 100% (95% CI: 73.5%–100%), specificity 94.8% (95% CI: 92.5–96.5%), positive predictive value 29.3% (95% CI: 17.2–45.0%), and negative predictive value 100% (95% CI: 99.3%–100%). Notably, events occurred only in patients with an LVEF 40%–50% and a history of ST-Elevation Myocardial Infarction.

**Conclusion:**

The PRESERVE EF study demonstrates that a simple, two-step, non-invasive risk factor-guided approach, followed by programmed ventricular stimulation, can effectively identify a subgroup of post-MI patients with preserved or mid-range LVEF ≥40 who are at high risk for major arrhythmic events.

**Clinical Trial Registration:**

Clinicaltrials.gov, identifier NCT02124018.

## GRAPHICAL ABSTRACT

**Figure:**
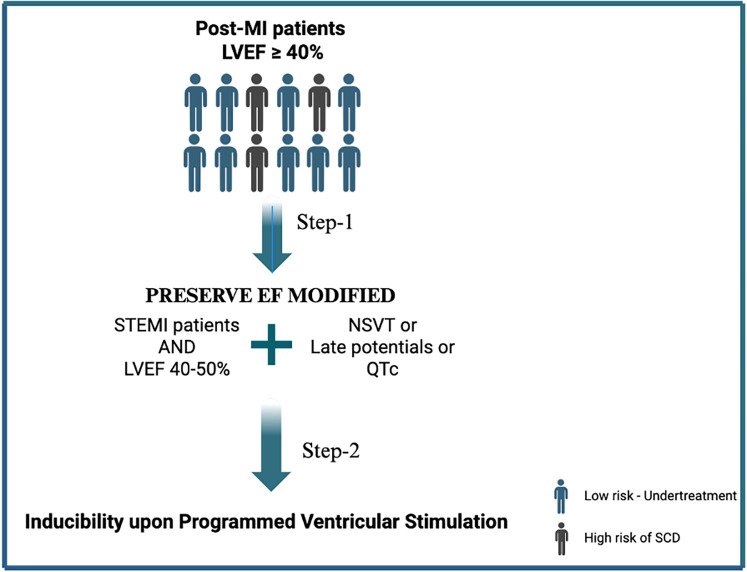

## Introduction

In contemporary clinical practice, the majority of post-myocardial infarction (MI) patients undergo primary revascularization, which generally preserves left ventricular ejection fraction (LVEF) (1). In these patients, the risk of sudden cardiac death (SCD) is relatively low; however, the vast size of this population in the era of primary Percutaneous Coronary Intervention poses a significant, unresolved clinical challenge (1). Despite the established inadequacy of LVEF as a sole criterion for risk stratification, current guidelines continue to rely primarily on this parameter (2–4).

In a previous study of post-MI patients with preserved LVEF, the annual incidence of SCD was calculated at 0.6% (5). Similar findings have been reported by the PROFID project, which highlighted the difficulty of predicting SCD in post-MI patients with mid-range or preserved LVEF using comprehensive multivariable models that include clinical variables, ECG parameters, biomarkers, and Cardiac Magnetic Resonance (CMR) imaging. While CMR-derived scar burden and fibrosis quantification have shown promise in identifying scar-related arrhythmogenesis, the PROFID analysis found limited incremental value of these imaging markers for SCD prediction in this specific population (6, 7). Nevertheless, the role of advanced imaging continues to evolve and may complement electrophysiological approaches in selected cases (8). Notably, this extensive effort did not take into consideration the only recent stratified approach with positive results in a population with preserved LVEF—the PRESERVE EF study. There is also growing interest in the role of electrophysiology study (EPS) for patients after myocardial infarction. This is highlighted by the ongoing PROTECT-ICD trial (NCT03588286), which is evaluating programmed ventricular stimulation (PVS) for risk stratification to guide early cardioverter-defibrillator implantation following an acute myocardial infarction (9, 10).

Hooks et al. demonstrated that ventricular tachycardia or ventricular fibrillation was the initial rhythm in approximately 50% of in-hospital cardiac arrests among patients with heart failure and preserved ejection fraction (HFpEF) (11). In a post-mortem study of 5,869 SCD victims from the Fingesture registry in Finland (12), the majority of patients showed evidence of myocardial scaring on autopsy, even though many had no documented clinical history of coronary artery disease prior to cardiac arrest. Remote silent myocardial infarction (MI) was evident in a substantial proportion of cases (42.5%). Moreover, the unrecognized presence of MI scarring was independently associated with an increased risk of sudden cardiac death (SCD) in the general population, along with the presence of triggering factors such as myocardial ischemia occurring during physical exercise (13).

The PRESERVE EF study (10) assessed the performance of a multifactorial, two-step, PVS-inclusive approach in identifying high-risk post-MI patients with LVEF ≥40% who are at increased arrhythmic risk and would benefit from implantable cardioverter-defibrillator (ICD). The approach proposed a two-step algorithm for risk stratification in post-MI patients with mid-range and preserved LVEF. In the first step, patients with at least one non-invasive risk factor (NIRF) were referred for EPS. In the second step, an ICD was implanted in patients with inducible sustained ventricular tachyarrhythmias during PVS. In this report, we provide an analysis of the 8-year follow-up outcomes of this stratification approach.

## Methods

### Trial design

The PRESERVE EF study is a multicenter, prospective, observational cohort study (clinicaltrials.gov identifier NCT02124018) conducted across seven cardiology departments in Greece. Short-term outcomes (mean follow-up, 32 months) have been reported previously (10). The trial was physician-initiated and received unrestricted funding from Medtronic Hellas S.A.; however, the sponsor did not provide support for the current analysis. The academic steering committee designed the trial protocol, which was approved by all relevant local review boards and endorsed by the Hellenic Society of Cardiology. An anonymized online database was created and maintained on the society’s servers (14). An independent data and safety monitoring board oversaw the trial.

### Participants

Eligible participants were post-angiographically proven MI patients, enrolled at least 40 days after the event (or 90 days after surgery if they underwent coronary artery bypass grafting) (15, 16). All patients had LVEF ≥40% (assessed after 40 or 90 days, respectively, from the index event), and were either revascularized or not—provided there was no evidence of active ischemia (following negative myocardial scintigraphy/exercise treadmill test/stress echocardiography in the previous 6 months). All participants were required to be on optimal tolerated medical therapy, which for the long-term outcomes analysis was defined according to the latest guidelines (17). The full list of inclusion and exclusion criteria is available in the study protocol (clinicaltrials.gov identifier NCT02124018) (14). All patients provided written informed consent.

### Study protocol

A two-step stratification algorithm was implemented (14). In the first step, ambulatory 24-h and signal-averaged electrocardiogram recordings were obtained and evaluated for the presence of NIRFs, defined as follows: (i) >30 premature ventricular complexes/hour on 24-h electrocardiography, (ii) presence of non-sustained ventricular tachycardia (NSVT) on 24-h electrocardiography, (iii) 2/3 positive criteria for late potentials, either conventional or modified, (iv) QTc derived from 24-h electrocardiography >440 ms (men) or >450 ms (women) according to the Fridericia formula from a signal recorded in three pseudo-orthogonal leads, (v) ambulatory T-wave alternans ≥65*μ*V in two Holter channels, (vi) standard deviation of normal RR intervals ≤75 ms on 24-h electrocardiography, (vii) deceleration capacity ≤4.5 ms, and (viii) heart rate turbulence onset ≥0% and heart rate turbulence slope ≤2.5 ms) (10, 18–20). In step two, in the presence of at least one NIRF, patients underwent invasive PVS and were classified as inducible if sustained monomorphic ventricular tachycardia, ventricular flutter, or polymorphic ventricular tachycardia was induced Stimuli were introduced at two right ventricular sites—apex and outflow tract—at two drive train cycle lengths (CLs)—550 and 400 ms—at each site. Up to three extrasystoles were introduced after the drive train at each drive train cycle length, with coupling intervals progressively shortened in 10-ms increments down to 200 ms or until refractoriness was reached, starting from the last extra systole. The arrhythmia induced was defined as sustained monomorphic ventricular tachycardia when a uniform morphology of QRS complexes with a rate between 120 and 220 b.p.m. was observed, while persisting for ≥30 s (or shorter, if termination was necessary due to hemodynamic instability). Faster rates of regular monomorphic ventricular tachycardia (≥220 b.p.m.) not permitting QRS complexes to be readily distinguished from T waves, and without deterioration toward fibrillation, were defined as ventricular flutter, but this was included in the monomorphic category. Polymorphic ventricular tachycardia was defined as the presence of constantly changing morphologies and axis, eventually degenerating into fibrillation (the detailed PVS protocol is provided in the parent study) (10). After completion of the study protocol, patients were stratified into three groups:

- Group 1: no NIRFs present—no invasive PVS performed.
- Group 2: at least one NIRF present—non-inducible upon PVS.
- Group 3: at least one NIRF present—inducible upon PVS.

An ICD was offered only to Group 3 patients.

Consistent with clinical trials supporting the use of prolonged detection intervals at higher rates to reduce unnecessary treatment of self-terminating arrhythmias (21), the ventricular tachycardia therapy was programmed with a cycle length of 330 ms and a number of intervals to detect set at 32. Fibrillation therapy cycle length was set to 270 ms and number of intervals to detect was set to 18/24. In devices with time programming, the same cycle lengths were used but intervals were set to 7 s for CLs in the 270–330 ms range and to 2.5 s for CLs <270 ms. Ventricular tachycardia therapy consisted of several attempts to perform antitachycardia pacing, followed by cardioversion at progressively increasing energy. High-energy shocks were administered to terminate ventricular fibrillation. In 32 cases, dual-chamber ICDs were inserted, while in the remaining five cases, a single-chamber device was chosen by both the primary and implanting physicians, after excluding the presence of bradyarrhythmic aberration on the EPS.

### Follow-up

The primary endpoint of the study was the occurrence of major arrhythmic events (MAEs), defined as a composite of SCD, documented sustained ventricular tachycardia or ventricular fibrillation, and appropriate ICD activation (antitachycardia pacing or shock). SCD was defined as death occurring within 1 hour of symptom onset, in the absence of any other identifiable cause. In patients with an ICD, appropriate device therapy for sustained ventricular arrhythmia was considered a major arrhythmic event. It is important to note that appropriate ICD therapies, while indicative of high arrhythmic risk, serve as a surrogate endpoint and do not necessarily equate to prevented sudden cardiac death, as some treated arrhythmias might have been hemodynamically tolerated or self-terminating. Death was considered non-sudden cardiac if occurring in the context of heart failure deterioration. All other deaths were classified as non-cardiac. Patients who received implants (Group 3) were followed prospectively every 6 months with device interrogation and clinical assessment. For patients who did not undergo programmed ventricular stimulation (Group 1: no NIRFs; and Group 2: NIRFs but non-inducible on PVS), as well as for the 52 patients who declined PVS, follow-up consisted of structured telephone interviews. No patients were lost to follow-up for the assessment of sudden cardiac death. However, detailed clinical follow-up regarding outcomes (e.g., rehospitalizations) was not systematically conducted for the non-ICD groups in this prespecified long-term analysis of the implanted patients. New acute coronary syndromes were reported in some patients in Groups 1 and 2 during the extended follow-up period; these cases required re-stratification according to the study protocol and were therefore not included in the current analysis of the original risk stratification performance. Sensitivity, specificity, and predictive values for the multifactorial, two-step, PVS-inclusive approach were calculated according to the primary endpoint.

## Results

**Figure 1:**
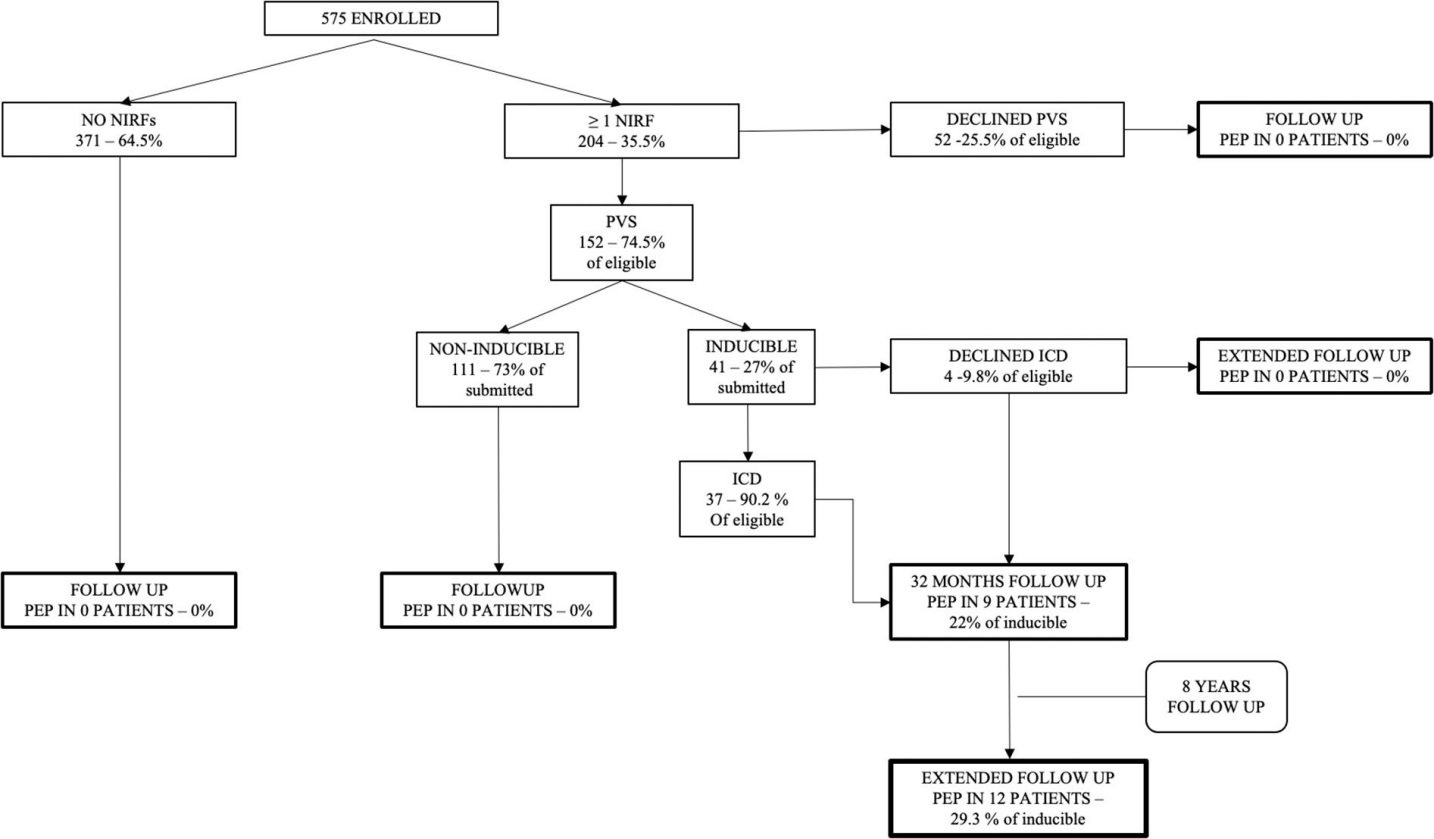
Long-term study flowchart. MAE, major arrhythmic event; NIRF, non-invasive risk factors; PEP, primary endpoint; PVS, programmed ventricular stimulation.

Between April 2014 and July 2018, a total of 575 patients were enrolled in this study. Over a median follow-up of 107 months (interquartile range: 98–114 months; range: 72–132 months) for the entire cohort of patients, no sudden cardiac deaths were observed outside the inducible group. Among the 37 patients who received an ICD, the median follow-up was 107 months (IQR: 99–115 months; range: 78–130 months). Figure 1 displays the flow of patients through the different steps of the study. Two-hundred and four patients (35.5%) had at least one NIRF. Fifty-two patients declined PVS, and four patients declined ICD implantation. There were no complications following PVS, and patients were usually discharged on the same day. There was a single case of pocket infection necessitating ICD extraction.

Out of these, 41 patients developed induced ventricular arrhythmia during the EPS. Ultimately, 37 patients (90.2% of the inducible patients) received an ICD. The majority of induced arrhythmias were monomorphic ventricular tachycardias (85.4%), with a short mean cycle length (244 ms). The characteristics of these patients are presented in Table 1, including comparisons of the baseline characteristics between patients with and without appropriate ICD activation during the 8-year follow-up.

**Table 1:** Demographic and clinical characteristics of the patients at baseline.

Variables	Group 1 (no NIRFs) (*N* = 371)	Group 2 (NIRFs, non-inducible) (*N* = 111)	Group 3 (NIRFs, inducible) (*N* = 41)	Patients with appropriate ICD activation (*N* = 12)	Patients without ICD activation (*N* = 29)
Age (years)	55.7 ± 10.2	60 ± 10.9	61.7 ± 9.2	63.8 ± 5.7	60.9 ± 10.1
Sex (% male)	84.7	86.5	97.6	100	96.6
Smoking (% yes)	59.8	52.3	53.7	58.3	51.7
Hypertension (% yes)	56.5	56	63.4	58.3	65.5
Dyslipidemia (% yes)	63.6	65.1	68.3	58.3	72.4
Diabetes mellitus (% yes)	14.9	15.6	36.6	41.7	34.5
LVEF (%)	45 (40–50)	45 (40–50)	45 (40–50)	45 (40–50)	45 (40–50)
LVEDD (mm)	53 ± 5.7	53 ± 5.7	53 ± 5.7	55 ± 7.1	52.5 ± 5.9
NYHA class I (%)	95.8	87.2	80.5	91.7	75.9
Type of infarction (% STEMI)	67.6	54.1	82.9	100	79.3
Follow-up duration (months)	106 ± 14.5	106 ± 14.5	106 ± 14.5	107.2 ± 14.6	105.7 ± 14.7
NIRF	Prevalence (%)				
LPs	0	31.5	51.2	83.3	37.9
PVCs (>30/h)	0	34.3	39	33.3	41.4
NSVT	0	23.1	46.3	66.7	37.9
QTc prolongation	0	40.4	36.6	50	31
Abnormal heart rate turbulence/deceleration capacity	0	9.3	9.8	0	13.8
Abnormal heart rate variability (SDNN <75 ms)	0	8.3	9.8	0	13.8
TWA > _65 μV	0	20.4	24.4	16.7	27.6

LP, late potentials; LVEDD, left ventricular end-diastolic diameter; LVEF, left ventricular ejection fraction; NIRF, non-invasive risk factor; NSVT, non-sustained ventricular tachycardia; PVC, premature ventricular complex; SDNN, standard deviation of normal-to-normal intervals; STEMI, ST-elevation myocardial infarction; TWA, T-wave alternans.

Table 2 indicates the characteristics of 12 patients with appropriate ICD activation. No inappropriate ICD activations were observed. Notably, in all patients with device activation, monomorphic ventricular tachycardia had been induced during PVS. Seven patients had more than one appropriate ICD activation, and four of these had more than two.

**Table 2:** Characteristics of patients with appropriate ICD activation.

Patients	Age	Infarction type	NYHA class baseline	NYHA class follow-up	LVEF baseline (%)	NIRFs present	Time after index MI (months) first episode	Induced arrhythmia cycle length (number of extra stimulus)	Clinical arrhythmia cycle length (ms)	Time after index MI (months) second episode	More than 2 episodes	Clinical arrhythmia therapy
1	64	STEMI	I	I	45	LPs	10.6	250 (2)	220	75	−	Shock
2	67	STEMI	I	I	47	LPs, PVCs, NSVT	11.3	240 (3)	250	−	−	Shock
3	63	STEMI	I	I	48	LPs, PVCs, NSVT	7	250 (2)	240	−	−	Shock
4	58	STEMI	I	II	45	LPs, QTc, NSVT	14	310 (2)	320	88	+	Shock
5	53	STEMI	I	II	45	LPs, PVCs, QTc, NSVT	12	300 (3)	300	65	+	Shock
6	59	STEMI	I	I	50	LPs, QTc, NSVT	47.7	248 (2)	310	−	−	ATP
7	70	STEMI	I	I	40	LPs, QTc, TWA	30.7	280 (2)	270	−	−	Shock
8	72	STEMI	I	III (new ACS)	50	NSVT	46.2	208 (3)	320	−	−	Shock
9	64	STEMI	II	II	50	QTc	59.5	260 (3)	260	72	+	Shock
10	59	STEMI	I	I	50	LPs	71	246 (3)	300	83	−	ATP
11	70	STEMI	I	I	50	LPs, PVCs, QTc, NSVT, TWA	82	280 (2)	270	83	−	Shock
12	61	STEMI	I	II	50	LPs, NSVT	23	240 (3)	270	35	+	Shock

ATP, antitachycardia pacing; ACS, acute coronary syndrome; LP, late potentials; LVEF, left ventricular ejection fraction; MI, myocardial infarction; NIRF, non-invasive risk factors; NSVT, non-sustained ventricular tachycardia; PVC, premature.

**Figure 2:**
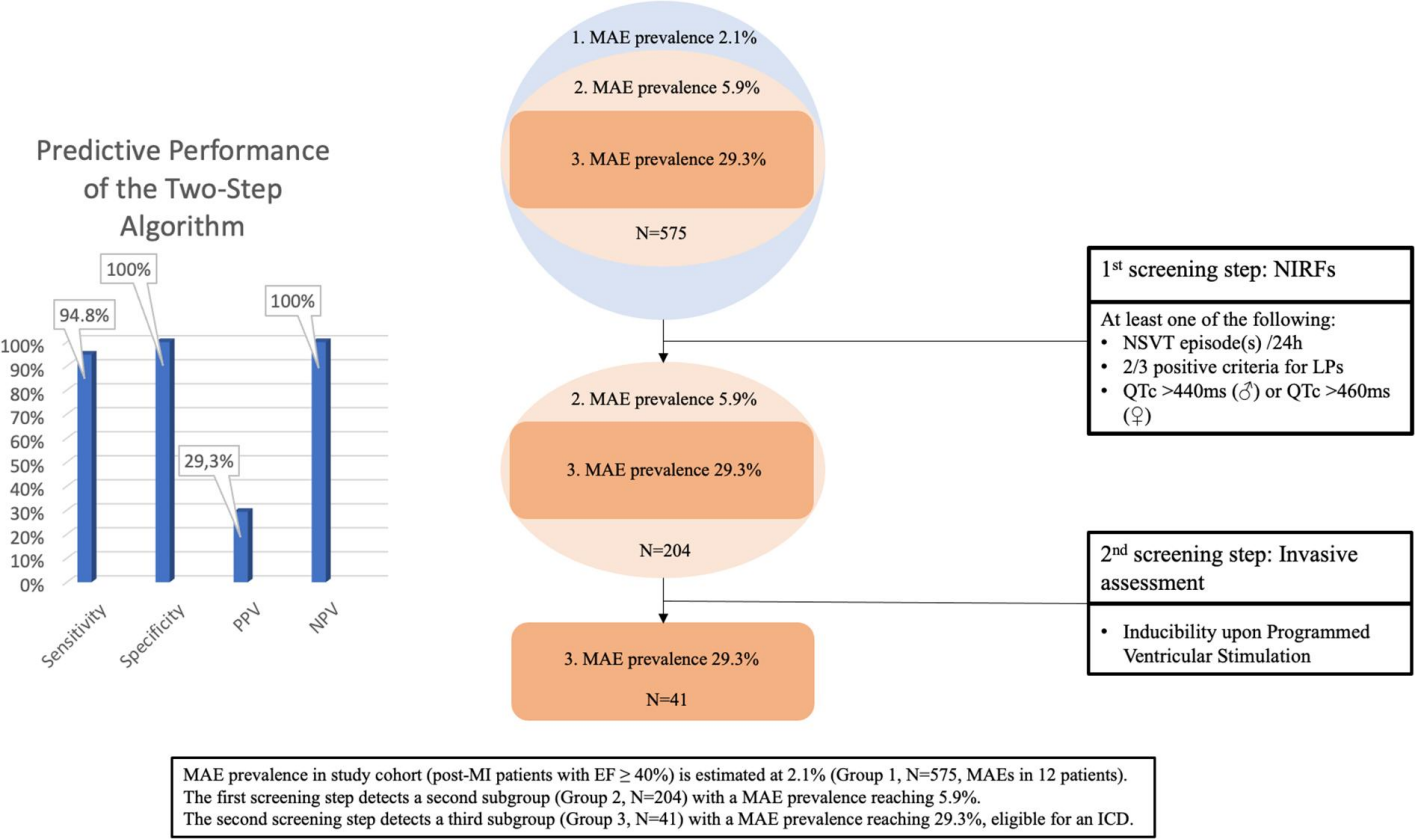
Updated study findings according to the long-term follow-up. LP, late potentials; MAE, major arrhythmic event; NIRF, non-invasive risk factors; NSVT, non-sustained ventricular tachycardia.

The updated (8.8-year follow-up) performance of the proposed approach (included both steps with NIRFs and PVS) was characterized by the following metrics: sensitivity 100% (95% CI: 73.5%–100%), specificity 94.8% (95% CI: 92.5–96.5%), positive predictive value 29.3% (95% CI: 17.2–45.0%), and negative predictive value 100% (95% CI: 99.3%–100%). The prevalence of major arrhythmic events in patients with ICD implantation was 29.3% (Figure 2). Notably, no events occurred in patients with LVEF > 50% (39.3% of total 575 patients) or in those without ST-Elevation Myocardial Infarction (33.7% of total 575 patients). Among NIRFs, late potentials, NSVT episodes, and QTc were the most commonly encountered non-invasive markers, and they can predict all ICD appropriate activation (Table 2). Applying these criteria to our database (LVEF 40%–50%, patients with STEMI, positive for late potentials, NSVT episodes, or QTc) yields a final sample of 29 patients eligible for ICD implantation after positive PVS, with an updated prevalence of 41.4%.

## Discussion

This 8-year follow-up analysis of the implanted patients of the PRESERVE EF trial demonstrated that, among post-MI patients with LVEF ≥40%, our multifactorial, two-step, PVS-inclusive approach effectively identified a high-risk subgroup that benefitted from ICD. The primary endpoint occurred in 12 out of 41 high-risk patients, all of whom had experienced STEMI, yielding a prevalence of 29.3% over a long-term follow-up period of 106 months. Notably, no sudden death events occurred among the study population without NIRFs (Group 1), nor among those with NIRFs who were non-inducible during PVS (Group 2). By redefining the inclusion criteria to patients with LVEF 40%–50%, STEMI, and the presence of late potentials, NSVT episodes, or QTc prolongation, the approach could reduce the number of patients requiring PVS while increasing the prevalence of major arrhythmic events in the selected high-risk cohort (41.4% in the present analysis). However, this simplified algorithm is derived from a retrospective subgroup analysis of the PRESERVE EF database and should be regarded as hypothesis-generating. Prospective validation in an independent cohort is required before any modification can be recommended for clinical practice.

Our findings indicate that the currently low annual incidence of sudden arrhythmic death in patients with preserved EF rises significantly among post-MI patients who are truly high risk and have inducible sustained ventricular tachyarrhythmias, along with the presence of at least one NIRF. The risk of receiving appropriate defibrillator therapy was substantial among those using defibrillators (22). The observed disparity in SCD rates between patients with LVEF ≤35% without ICDs and those receiving appropriate ICD interventions highlights the complexity of arrhythmic risk management and may be attributed to various factors. It is widely recognized that not all arrhythmias treated by defibrillators would be lethal if left untreated. To mitigate the risks of inappropriate activation and unnecessary interventions, ICD programming in this study required a relatively long detection interval in two zones: one between 180 and 220 bpm and the other above 220 bpm, consistent with relevant trials (21). This approach minimizes the risk of potentially harmful overtreatment.

The goal of the study was to develop a risk stratification scheme that would be low cost and widely accessible for any cardiologist in outpatient settings. Therefore, the first step of our approach reflects these characteristics. Notably, a modified version of the study, focused on fewer NIRFs, demonstrated that our method could be readily adopted in routine practice, enhancing awareness of SCD risk in this patient population. By refining the inclusion criteria (LVEF 40%–50%, STEMI, presence of late potentials, NSVT episodes, or QTc prolongation), the approach reduces the number of patients requiring PVS while simultaneously increasing the SCD prevalence among the selected cohort, thus lowering costs and avoiding unnecessary ICD implantation (23, 24). Cardiac magnetic resonance imaging was not evaluated in the present study. Although CMR assessment of scar size and border zone heterogeneity has demonstrated value in risk stratification for ventricular arrhythmias in several studies, particularly in patients with reduced ejection fraction or non-ischemic cardiomyopathy, its incremental benefit in post-MI patients with preserved LVEF was limited in the recent PROFID pooled analysis. The PRESERVE EF approach offers the advantage of being inexpensive, widely accessible in outpatient settings, and based on simple non-invasive electrocardiographic markers followed by a relatively straightforward electrophysiological study. Future research should explore whether combining electrophysiological testing with selective use of CMR could further refine risk prediction (8, 25).

Several limitations of the present study should be acknowledged. First, this was an observational analysis without a randomized control group. Second, the primary endpoint relied heavily on appropriate ICD therapies as a surrogate for major arrhythmic events. Although appropriate device activations are widely accepted as clinically relevant indicators of high arrhythmic risk, they are not synonymous with sudden cardiac death. Some of the treated ventricular arrhythmias may have been non-lethal or self-terminating in the absence of ICD. Therefore, the observed event rate may overestimate the true risk of sudden cardiac death in this population. A randomized controlled trial with a hard clinical endpoint (e.g., sudden cardiac death or all-cause mortality) would be required to definitively assess the benefit of ICD implantation in the high-risk subgroup identified by the PRESERVE EF algorithm. Furthermore, we cannot exclude the possibility of a new silent acute coronary syndrome during the 8-year follow-up. Given the study's focus on the long-term outcomes of the parent cohort, other NIRFs such as unexplained syncope (26, 27) were not evaluated in the first step of the algorithm. Cardiac magnetic resonance was also not evaluated in the parent study, but it correlated with high cost and poor results in the PROFID study (7, 28). Follow-up data for the entire cohort of 575 patients are not presented here in full, because new acute coronary syndrome cases occurred in some patients in Groups 1 and 2 during the extended follow-up period. Re-stratification of these patients would warrant a separate dedicated study. For the assessment of the primary endpoint (major arrhythmic events), telephone follow-up was successfully completed for all patients, with no cases of sudden cardiac death reported in Groups 1 and 2. Nevertheless, we acknowledge that surveillance intensity was higher in the ICD group (regular device interrogations) compared with telephone contacts in the non-ICD groups. This difference in follow-up intensity represents a potential limitation when interpreting the perfect negative predictive value of the proposed algorithm.

It should be noted that for patients with preserved LVEF, no randomized clinical trial for risk stratification currently exists, while for patients with reduced LVEF, available studies are outdated. In this context, the PROFID project's decision to limit its trial to patients with LVEF ≤35% (NCT05665608) is justified. This reflects the impact of contemporary pharmacological treatment of heart failure, which has been shown to reduce SCD (29). Consequently, many patients with LVEF ≤35% may receive ICDs without deriving a survival benefit.

## Conclusion

Our advanced risk stratification strategy, derived from the PRESERVE EF study, may play a useful role in arrhythmic risk assessment among post-myocardial infarction patients with LVEF ≥40%. However, because this was an observational study without a control group of high-risk patients who did not receive an ICD, a definitive survival benefit cannot be claimed. Until a randomized clinical trial provides evidence of improved survival with ICD therapy in this population, the PRESERVE EF approach—with or without modification using fewer NIRFs—remains one of the very few prospectively evaluated risk stratification strategies available for post-MI patients with preserved ejection fraction (Graphical Abstract).

## Data Availability

The raw data supporting the conclusions of this article will be made available by the authors without undue reservation.
